# Spontaneous Pneumo-Mediastinum in a Post-COVID-19 Patient with Systemic Sclerosis

**DOI:** 10.3390/healthcare10030529

**Published:** 2022-03-14

**Authors:** Ilaria Mormile, Mauro Mormile, Gaetano Rea, Angelica Petraroli, Vittoria Barbieri, Amato de Paulis, Francesca Wanda Rossi

**Affiliations:** 1 Department of Translational Medical Sciences, University of Naples Federico II, 80131 Naples, Italy; ilariamormile@virgilio.it (I.M.); ambulatoriopetraroli@gmail.com (A.P.); francescawanda.rossi@unina.it (F.W.R.); 2 Department of Clinical Medicine and Surgery, University of Naples Federico II, 80131 Naples, Italy; mormile@unina.it; 3 Department of Radiology, “Vincenzo Monaldi” Hospital-AORN Ospedale Dei Colli, 80131 Naples, Italy; gaetano.rea71@gmail.com; 4 Center for Basic and Clinical Immunology Research (CISI), WAO Center of Excellence, University of Naples Federico II, 80131 Naples, Italy; 5 Post-Graduate Program in Clinical Immunology and Allergy, University of Naples Federico II, 80131 Naples, Italy; tarta.ruga1991@hotmail.it

**Keywords:** interstitial lung disease, pneumo-mediastinum, post-COVID-19 syndrome, pulmonary emphysema, systemic sclerosis

## Abstract

Pulmonary involvement is the most common cause of death among patients with systemic sclerosis (SSc). The current coronavirus disease 2019 (COVID-19) is particularly problematic to manage in SSc patients since they may experience a more severe evolution of COVID-19 due to the pre-existent interstitial lung disease (ILD) and the administration of immunosuppressive treatments. In addition, the remarkable radiological similarities between SSc-ILD and COVID-19 complicate the differential diagnosis between these two entities. Herein, we present the first case of spontaneous pneumo-mediastinum in a post-COVID-19 patient with SSc. In our patient, both smoking and pulmonary fibrosis could lead to cyst formation, which possibly spontaneously broke and caused pneumo-mediastinum. Moreover, megaesophagus perforation due to the smooth muscle atrophy, replacement with fibrosis, and achalasia may extend into the mediastinum or pleural space and has also been described as a rare case of spontaneous pneumo-pericardium. Finally, spontaneous pneumo-mediastinum and pneumothorax have been recently reported as an established complication of severe COVID-19 pneumonia and among COVID-19 long-term complication. This case report underlines that the worsening of respiratory symptoms in SSc patients, especially when recovered from COVID-19, requires further investigations for ruling out other tentative diagnoses besides the evolution of the SSc-ILD.

## 1. Introduction

Systemic sclerosis (SSc) is a chronic autoimmune disease characterized by immune inflammation, vasculopathy, and enhanced fibrosis [1]. Pulmonary involvement is a common finding in SSc, occurring in up to 70–90% of cases and can be due to parenchymal and vascular disorders [2]. Interstitial lung disease (ILD) and pulmonary hypertension are frequent manifestations. Clinically significant ILD occur in up to 40% of cases, although up to 90% of SSc patients may have radiological or post-mortem evidence of ILD [3]. Common high-resolution computed tomography (HRCT) findings are ground-glass opacities, peripheral reticulations, and consolidations [4,5]. Many different histologic patterns have been described, fibrotic nonspecific interstitial pneumonia (NSIP) being the most prevalent [6], followed by cellular NSIP, usual interstitial pneumonia (UIP), organizing pneumonia, lymphoid hyperplasia, and in rarer occasions non-necrotizing granulomas [3,6]. Pulmonary hypertension is the second most common pulmonary manifestation, occurring in 20% of cases [3]. It significantly worsens the SSc patient’s long-term prognosis [7]. Pleural irregularities associated with the ILD are frequently described in SSc patients [3], despite that pleural involvement, including pleural effusion and pneumothorax, are a quite rare complication [8,9]. Pneumothorax and pneumo-mediastinum are ILD complications mainly due to the rupture of subpleural cysts in patients with underlying ILD [3,10,11]. Pneumothorax has to be considered a potentially life-threatening complication since re-expansion of the lung is slow, and it is associated with a poorer prognosis [11].

The current coronavirus disease 2019 (COVID-19) is a global health and economic emergency which is particularly problematic to manage in SSc patients since it may significantly complicate the pre-existent pulmonary clinical scenario [12]. CT examination has shown high sensitivity in diagnosing COVID-19 [13,14]. The Dutch Radiological Society created a COVID-19 Reporting and Data System (CO-RADS) [15], which provides a level of suspicion for pulmonary involvement of COVID-19 based on the features seen on a non-enhanced chest CT. The level of suspicion increases from very low (CO-RADS 1) to very high (CO-RADS 5). CO-RADS 1 implies a very low suspicion for pulmonary involvement by COVID-19 based on either a normal CT or radiological findings of unequivocal non-infectious etiology (i.e., mild or severe emphysema, perifissural nodules, lung tumors, or fibrosis) [15,16]. CO-RADS 2 means a low level of suspicion for pulmonary lesion resulting from COVID-19 based on CT findings in the lungs that are typical of infectious etiology that are considered not compatible with COVID-19 (e.g., bronchitis, infectious bronchiolitis, bronchopneumonia, lobar pneumonia, and pulmonary abscess). Findings include a tree-in-bud sign, a centrilobular nodular pattern, lobar or segmental consolidation, and lung cavitation [15,16]. CO-RADS 3 is characterized by findings equivocal for pulmonary involvement of COVID-19 that can also be seen in other types of viral pneumonia or non-infectious etiologies such as perihilar ground-glass, extensive homogenous ground-glass with or without sparing of some secondary pulmonary lobules, ground-glass together with smooth interlobular septal thickening with or without pleural effusion, and small ground-glass opacities that are not centrilobular or not located close to the visceral pleura [15,16]. CO-RADS 4 implies a high suspicion of pulmonary involvement by COVID-19 with typical CT findings but with some overlap with other types of viral pneumonia. Findings included in CO-RADS 4 are similar to CO-RADS 5 but not located in contact with the visceral pleura or located strictly unilaterally, have a predominantly peribronchial distribution, or superimposed on severe diffuse pre-existing pulmonary abnormalities [15,16]. CO-RADS 5 means a very high level of suspicion for pulmonary lesion resulting from COVID-19, based on typical CT findings. Obligatory features are ground-glass opacities, with or without consolidations, close to visceral pleural surfaces, including the fissures, and a multifocal bilateral distribution [15,16]. CO-RADS 6 indicates COVID-19 infection proven by positive RT-PCR test for virus-specific nucleic acid [15].

Although CT examination has shown high sensitivity in diagnosing COVID-19 [13,14] the differential diagnosis may be complicated in patients with concomitant SSc as SSc-ILD may mask or mimic early COVID-19 lesions [17]. Herein, we present the first case of spontaneous pneumo-mediastinum in a post-COVID-19 patient with SSc.

## 2. Case Description

**Figure 1:**
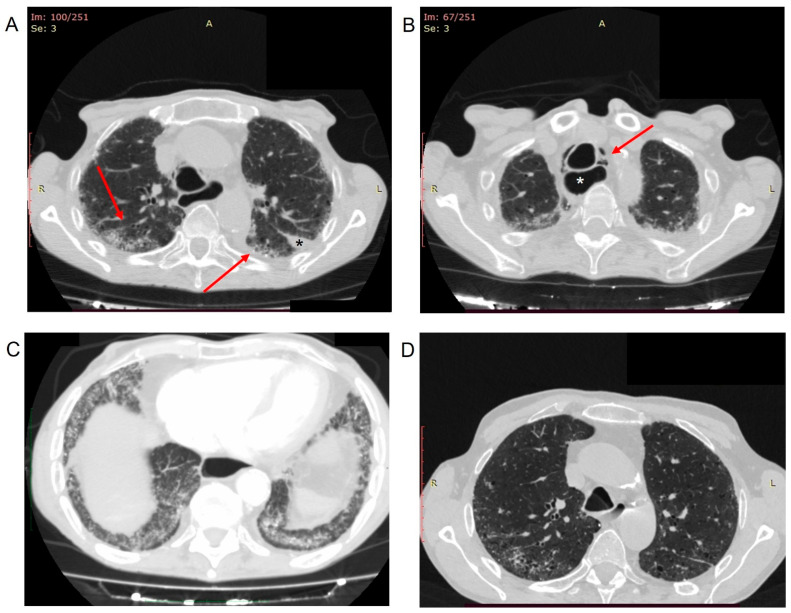
(**A**–**D**) Post-COVID-19 computed tomography (CT) pulmonary angiography. (**A**) Diffuse fibrotic changes with subpleural ground-glass opacity (*arrows*), traction bronchiectasis, and several emphysematous bullae were evident. Mild pleural effusion in the left oblique fissure (*black asterisk*) and mild left-sided paravertebral pleural effusion. (**B**) Pneumo-mediastinum (*arrow*) with enlargement of the esophagus (*white asterisk*). (**C**) Mild ground-glass opacification in both lower lobes with sparing of subpleural regions. (**D**) CT-Scan of the thorax performed two months before the COVID-19, showing peripheral interstitial thickening and traction bronchiectasis due to the systemic sclerosis interstitial lung disease.

A 65-year-old ex-smoker male with rapidly progressive SSc and ILD accessed our Immunology Clinic presenting severe breathlessness and a dry cough. He had recovered from COVID-19 pneumonia nine months earlier. The infection presented with anosmia, fatigue, headache, low-grade fever, and dyspnea. The overall severity of symptoms was mild, not requiring hospitalization. Therefore, the infection had been treated at home with high doses of oral glucocorticoids (prednisone 50 mg/day for two weeks) with subsequent prednisone tapering to the maintenance dose of 10 mg/day, ceftibuten 400 mg/day, and low molecular weight heparin (LMWH) 4000 UI/day, without invasive or non-invasive ventilation. When the patient was referred to our care, he was taking prednisone 10 mg/day and mycophenolate mofetil 2 g/day as a maintenance therapy. An extensive skin thickening of the face, upper and lower limbs associated with contractures of the fingers, and elbow ulcers were evident on physical examination. The patient’s height was 169 cm, and his weight was 44 kg (body mass index 15.4 kg/m^2^). He had blood pressure of 110/60 mmHg, pulse 108/min, and respiration rate at rest 25/min. His body temperature was within normal limits. Fine bibasilar inspiratory crackles and diffuse rhonchi, in the absence of mediastinal crepitus, were found on chest examination. Laboratory investigations revealed an increase in D-dimer (2383 ng/mL; reference range 0–500), fibrinogen concentration (396 mg/dL, reference range 160–350), and N-terminal fragment brain natriuretic peptides (NT-pro-BNP, 413 pg/mL; reference range <125). Increased level of leucocytes (12.18 × 103/µL; reference range 4.8–10.8), neutrophils (9.61 × 103/µL; reference range 1.8–7), C-reactive protein concentration (1.96 mg/dL; reference range <0.5), and severe acute respiratory syndrome coronavirus 2 (SARS-CoV-2) IgG antibodies (>400 AU/mL; reference range < 18) were also found. Hemoglobin, platelet count, blood chemistry, erythrocyte sedimentation rate, IgG, IgA, IgM, C3, C4, urine analysis, parathyroid hormone, vitamin D dosage, and tumor markers levels (AFP, CEA, CA 19-9, CA 15-3, CA 125, β2 microglobulin, and prostate-specific antigen) were within normal limits. The arterial blood gas (ABG) analysis showed PH 7.39 (reference range 7.35–7.45), PaO_2_ 46.1 mmHg (reference range 75–100 mmHg), PaCO_2_ 57.6 mmHg (reference range 35–45 mmHg), and oxygen saturation 83.1% on room air. Pulmonary function tests showed a restrictive pattern at the spirometry and a decrease of the diffusing capacity of the lung for carbon monoxide (DLCO). Doppler echocardiography was performed: pulmonary arterial pressure was severely increased (55 mmHg) with right atrial and ventricular dilatation. Tachycardia with ventricular premature beats was evident on the electrocardiogram (EKG). Considering the worsening of respiratory symptoms and the nearly fourfold rise in D-dimer level, the patient underwent a computed tomography (CT) pulmonary angiography, which showed no pulmonary embolism at the main pulmonary artery, and at its lobar, segmental, and sub-segmental pulmonary branches on both sides, but with a non-homogeneous opacification of the sub-segmental distal branches of the middle and inferior lobes. In addition, fibrotic changes, traction bronchiectasis, and several emphysematous bullae were evident (Figure 1A). Free air in the anterior mediastinum extended up to the base of the neck (pneumo-mediastinum) with generalized enlargement of the esophagus was also found (Figure 1B). In addition, mild ground-glass opacification in both lower lobes with sparing of subpleural regions were evident (Figure 1C).

Given the diagnosis of megaesophagus with a history of achalasia, CT-Scan of the thorax with oral contrast administration was performed, ruling out a spontaneous perforation of the esophagus. The CT scan images were compared with CT scans of the thorax performed two months before the COVID-19 (Figure 1D), showing a worsening of the ILD. The patient was managed with a conservative treatment strategy of the pneumo-mediastinum and treated with methylprednisolone 40 mg/day intravenously, LMWH 4000 UI given every twelve hours subcutaneously, 4 g piperacillin/0.5 g tazobactam every eight hours intravenously, one inhalation of fluticasone propionate/salmeterol combination 250/50 µg twice daily, one inhalation tiotropium 55 µg once daily, and oxygen therapy, with clinical improvement. Gradually his general condition improved, and a follow-up CT scan showed resolution of pneumo-mediastinum.

## 3. Discussion

Pulmonary involvement is the most common cause of death among patients with SSc [18]. Spontaneous pneumo-mediastinum has occasionally been reported in patients with SSc (Table 1) [11,19,20,21], in one case occurring after pulmonary function testing [22].

Most of the patients presented with acute dyspnea [11,19,20,21], but subacute dyspnea [10] and asymptomatic course [23] are also possible. Pneumo-mediastinum and pneumothorax are often due to the rupture of subpleural cysts [19]. In addition, these two complications may occur as a rare consequence of perforations of the gastrointestinal tract [24]. Indeed, megaesophagus perforation due to the smooth muscle atrophy, replacement with fibrosis, and achalasia may extend into the mediastinum or pleural space and has been described as a rare case of spontaneous pneumo-pericardium [24].

In our patient, both smoking and pulmonary fibrosis may lead to cyst formation, which possibly spontaneously broke and caused pneumo-mediastinum. However, to the best of our knowledge, this is the first case of spontaneous pneumo-mediastinum in a post-COVID-19 patient with SSc. Spontaneous pneumo-mediastinum and pneumothorax have been reported as an established complication of severe COVID-19 pneumonia, possibly due to diffuse alveolar injury leading to alveolar rupture and air leak [25]. In addition, delayed recurrent spontaneous pneumothorax, presenting four weeks after recovery from COVID-19, has recently been described and should be considered as a COVID-19 long-term complication together with pulmonary thromboembolism [25]. The current knowledge on the full range of symptoms and risk factors of the so-called “post-COVID syndrome” or “Post-Acute Sequelae of SARS-CoV-2 infection (PASC)” is still limited due to the paucity of long-term follow-up data, especially among individuals with mild COVID-19. Approximately 80% of hospitalized patients with COVID-19 show persistent symptoms several months after infection onset [26,27]. However, our patient presented with a COVID-19 infection that did not require hospitalization, which opens up a point of reflection on long-term outcomes among individuals with mild COVID-19. A considerable portion of low-risk individuals with mild COVID-19 exhibit a diversity of long-term symptoms (e.g., anosmia, ageusia, fatigue, headache, muscle/joint pain, and dyspnea), which may disrupt work, social, and home life [26]. Given that, the concomitance of comorbidities might represent an additional risk factor for long-term sequelae of COVID-19 [28,29]. Despite the relatively mild symptoms experienced by our patient during the acute COVID-19 infection, his post-COVID-19 interstitial CT-scan pattern was significantly worsened than the previous one (Figure 1A,D). This condition raises the question of whether these findings were due to SSc-ILD, COVID-19 pneumonia, or their coexistence. SSc patients may experience a more severe evolution of COVID-19 due to the pre-existent ILD and the administration of immunosuppressive treatment [17]. The occurrence of COVID-19 pneumonia may inevitably complicate the multifaceted pulmonary scenario found in SSc since it is characterized by interstitial involvement with radiological features similar to SSc-ILD [17]. In addition, patients with chronic ILD may be more prone to develop a severe COVID-19 lung infection [13]. In addition, the remarkable radiological similarities between SSc-ILD and COVID-19 (i.e., the presence in both diseases of bilateral and subpleural ground-glass opacities, with or without consolidations [30]) complicate the differential diagnosis between these two entities [17]. A recent multicentric study analyzing CT features of 52 patients with COVID-19 and 47 patients with SSc-ILD evaluated the main CT features related to both diseases, identifying the specific lesions that could help in differential diagnosis [30]. The authors observed that the presence of consolidation in the lower lobes might suggest COVID-19 pneumonia, while the presence of fibrosis inside ground-glass opacities may indicate SSc-ILD [30]. However, while distinguishing between COVID-19 and SSc-ILD may be easier when they occur alone, the scenario may be significantly complicated when these two conditions coexist. For instance, during the early phases of COVID-19, consolidations can be absent, and ground-glass opacities may be the only CT feature [30]. Moreover, the clinical presentation could also be similar in COVID-19 pneumonia and SSc-ILD [18,30,31,32]. Indeed, although the rapid onset of dyspnea and fever could point towards SARS-CoV-2 infection, fever may be absent in patients with autoimmune disease due to immunosuppressive treatment [31,32,33].

Analyzing the evolution of respiratory and radiological features following the COVID-19 infections in patients with autoimmune diseases should be particularly relevant since a significantly higher prevalence of COVID-19 is observed in a large series of patients with systemic autoimmune diseases than the general population [34]. In addition, it has been observed that COVID-19 can exacerbate or cause the onset of many autoimmune diseases by triggering autoantibody production in genetically predisposed patients [35]. Several cases of possible new onset of autoimmune diseases such as systemic lupus erythematosus [36,37,38], Guillain Barre Syndrome [39], and SSc [35] have been described in the medical literature. Molecular mechanisms underlying the complex link between COVID-19 and autoimmunity are still unknown. Current hypotheses include molecular mimicry due to the immune cross-reaction between epitopes and host antigens, an increase in interferon and other cytokines production leading to the disruption of immune tolerance and defect of the function of dendritic cells [35,40].

## 4. Conclusions

This case report underlines that COVID-19 management could be particularly problematic in patients with SSc even after the resolution of the acute phase. The worsening of respiratory symptoms in patients with SSc recovered from COVID-19 pneumonia requires further investigations to assess the potential evolution of the lung damage and rule out other tentative diagnoses such as pneumo-mediastinum besides the evolution of the SSc-ILD.

## Figures and Tables

**Table 1:** Pneumo-mediastinum cases in systemic sclerosis (SSc) patients reported in the literature.

References	Total SSc Patients (*n*)	Cause	Outcome
[10]	1 ^a^	Spontaneous	Progressive hypoxia requiring intubation and complicated by *Klebsiella pneumonia* and renal failure
[19]	1	Spontaneous	Resolution with conservative treatment strategy
[23]	1	Spontaneous	Resolution with conservative treatment strategy
[22]	1	Pulmonary function testing	Resolution with conservative treatment strategy
[20]	1	Spontaneous	Resolution with conservative treatment strategy
[21]	1	Spontaneous	Resolution with conservative treatment strategy
Present case	1	Spontaneous	Resolution with conservative treatment strategy

^a^ Patient with limited cutaneous systemic sclerosis and myositis overlap.

## Data Availability

All datasets generated for this study are included in the article.
